# Case report: Necrotizing fasciitis involving the bilateral scrotum and right leg caused by *Streptococcus pyogenes* in a healthy male: a multidisciplinary diagnostic and therapeutic analysis

**DOI:** 10.1186/s12879-026-12889-4

**Published:** 2026-02-17

**Authors:** Xiaohua Li, Jianbo Xin, Wuyunbilige Bao, Yu Fu

**Affiliations:** 1 Department of Clinical Laboratory, Ordos Central Hospital, Ordos, Inner Mongolia China; 2 Department of Burn and Plastic Surgery, Ordos Central Hospital, Ordos, Inner Mongolia China; 3 Intensive Care Unit (ICU), Ordos Central Hospital, Ordos, Inner Mongolia China

**Keywords:** Necrotizing fasciitis, *Streptococcus pyogenes*, Multidisciplinary communication, Case report

## Abstract

**Introduction:**

Necrotizing fasciitis (NF) following minor trauma in healthy individuals is rare and poses a considerable diagnostic challenge. Due to its nonspecific early presentation, NF is associated with high rates of misdiagnosis and mortality, underscoring the critical need for early recognition and intervention.

**Case presentation:**

A previously healthy 37-year-old male developed rapidly progressive necrotizing fasciitis of the scrotum and right lower extremity following minor bicycle-related abrasions. Upon admission, the patient presented with a high-risk Laboratory Risk Indicator for Necrotizing Fasciitis (LRINEC) score of 8, and imaging studies demonstrated rapid spread of infection from the right inguinal region to the right lower extremity and perineum, with subsequent progression to septic shock and multiple organ dysfunction syndrome. Urgent multidisciplinary team (MDT) collaboration is critical. Through emergency fasciotomy and identification of Group A Streptococcus (GAS) infection, the diagnosis of NF was definitively established. Combined with high-dose penicillin G, and comprehensive supportive measures, he achieved complete recovery without sequelae at 6-month follow-up.

**Conclusions:**

Type II NF related with GAS can even occurred in low-risk populations. Early diagnosis relies on LRINEC scoring combined with imaging. Time-critical MDT with immediate coordination for pathogen identificationis crucial for survival.

**Clinical trial number:**

Not applicable.

## Introduction

Necrotizing Fasciitis (NF) is a life-threatening, rapidly progressive soft tissue infection involving the superficial fascia and adjacent structures, with a mortality rate exceeding 20% [1]. When NF affects the perineal, genital, or perianal regions, it is classified as Fournier’s gangrene (FG) [2]. The disease frequently affects individuals with predisposing conditions such as diabetes mellitus, immunosuppression, or chronic alcoholism. FG is commonly polymicrobial in nature, often originating from dermal, urogenital, or colorectal sources [3]. Though Group A Streptococcus (GAS) is a well-established primary pathogen Western series, accounting for 31%-66.6% of NF cases [4–8]. The incidence of monomicrobial GAS-induced NF in China is rare [9, 10].

Diagnosis remains a significant clinical challenge, as the early presentation of NF is often nonspecific, mimicking cellulitis or other benign soft-tissue infections. Consequently, misdiagnosis rates are reported to be as high as 50%–75% [11]. NF carries a mortality rate of 20–30%, and emergency surgical intervention is mandatory upon diagnosis [12]. We herein report a rare and instructive case of type II FG caused by monomicrobial GAS in a previously healthy, immunocompetent adult following minor trauma, aiming to highlight the diagnostic pitfalls and underscore key management principles.

## Case presentation

### History and clinical findings

A 37-year-old Chinese male with no history of diabetes or immunosuppression presented to the hospital on the night of March 21, 2022, with a one-day history of progressive bilateral scrotal pain and swelling. His symptoms began on March 20 as scrotal discomfort following a bicycle ride, which resulted in minor superficial abrasions. This incident was preceded by alcohol consumption and prolonged lateral recumbency the previous day. The patient reported no recent history of fever, sore throat, or upper respiratory symptoms. He was initially misdiagnosed with testicular torsion based on ultrasound findings at a local hospital and received no specific intervention. As his symptoms rapidly worsened, characterized by a marked increase in scrotal size, escalating pain, and noticeable skin discoloration progressing to purple. Then, he was admitted to the hospital for further management.

**Fig. 1:**
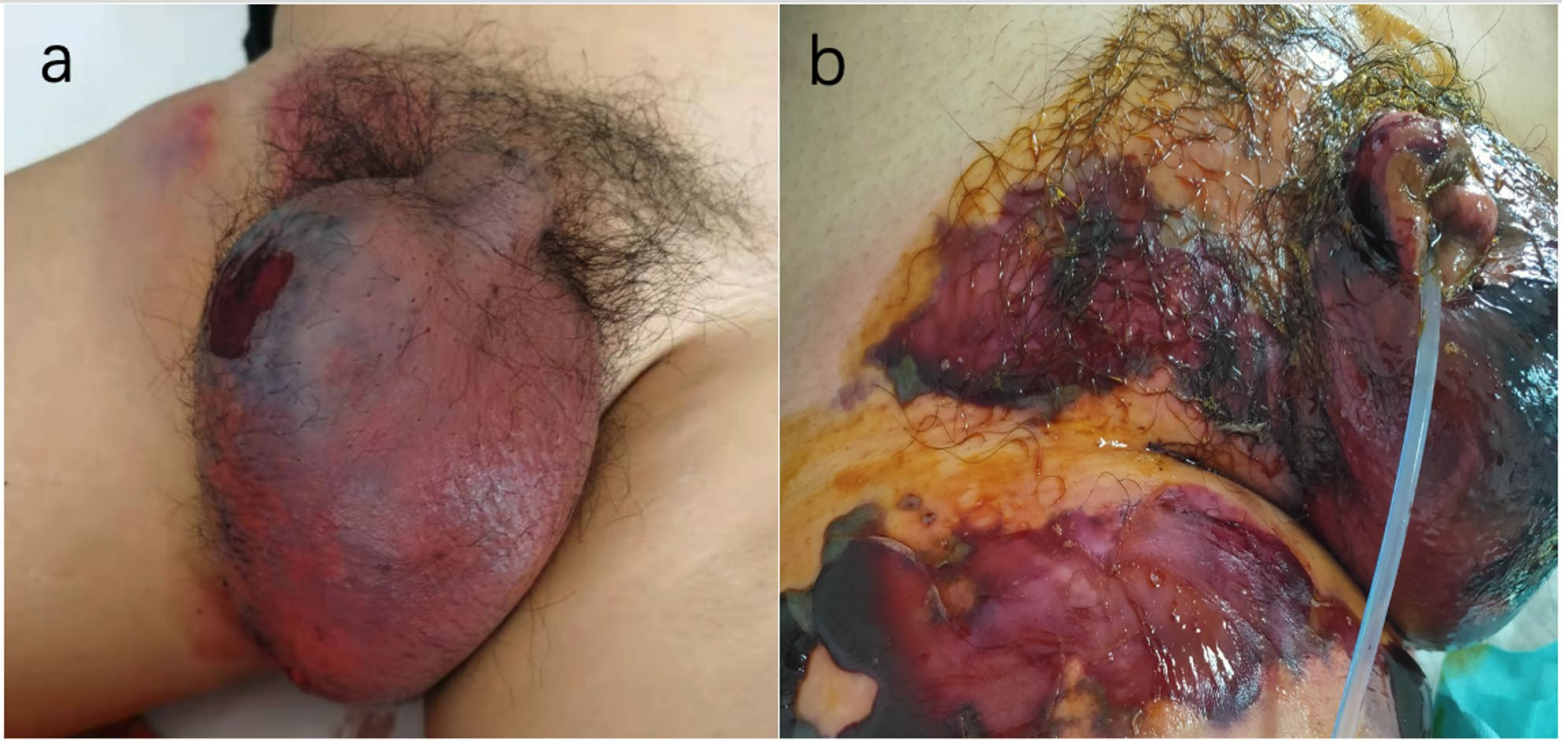
Patient’s skin lesions of the admission and the next day

On admission, his vital signs were as follows: body temperature, 36.3 °C; heart rate, 117 beats/min; and blood pressure, 110/82 mmHg (BMI 29). Physical examination revealed bilateral scrotal erythema with purple discoloration, severe tenderness, and significant swelling (Fig. 1a), with intense pain extending to the right medial thigh.

### Diagnostic work-up

Laboratory investigations showed significant systemic inflammation: leukocytosis (white blood cell count 12.43 × 10⁹/L, reference range: 3.5–9.5 × 10⁹/L) with neutrophils (91.4%, reference range: 50–70%), elevated C-reactive protein (CRP 282.74 mg/L, reference range: 0–6 mg/L), and pro-calcitonin (PCT 59.32 ng/mL, reference range: 0–0.046 ng/mL). Electrolyte and renal function abnormalities included hyponatremia (134.9 mmol/L, reference range: 137.0–147.0 mmol/L) and acute kidney injury (serum creatinine 199 µmol/L, reference range: 59–104 µmol/L). These findings resulted in a Laboratory Risk Indicator for Necrotizing Fasciitis (LRINEC) score of 8, placing the patient in a high-risk category for NF [13, 14]. This classification was based on elevated CRP ≥ 150 mg/L (4 points), hyponatremia (2 points) and elevated creatinne (2 points). A throat culture was not performed during his admission.

**Fig. 2:**
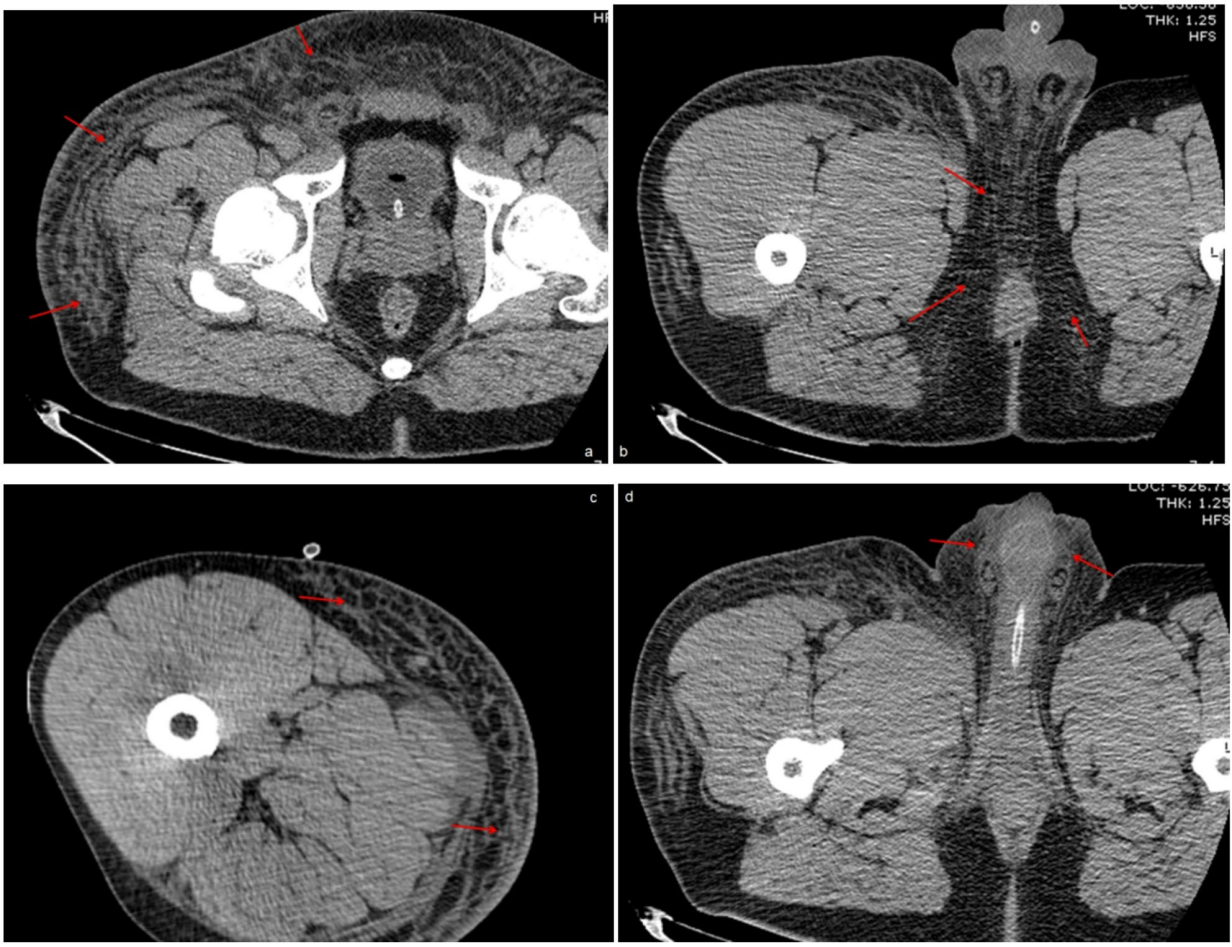
CT imaging showing subcutaneous soft tissue edema in the right hip, pelvic floor, and medialright thigh

Ultrasound revealed soft tissue thickening in the right inguinal region with scrotal wall edema. An initial computed tomography (CT) scan demonstrated extensive subcutaneous edema and soft tissue thickening extending from the right hip to the pelvic floor and medial right thigh (Fig. 2).

### Therapeutic course and outcome

On the night of admission, a misdiagnosis as “scrotal edema” led to an ineffective regimen of empirical anti-inflammatory therapy with amikacin, dexamethasone and parecoxib sodium. The next day, a dermatology consultation misidentified the condition as drug-induced dermatitis (Fig. 1b), resulting in treatment with methylprednisolone (40 mg daily) and topical Compound Huangbai Lotion, which failed to control disease progression.

At this point, preliminary results from wound smears raised suspicion for a infection. A consultation with the Department of Critical Care Medicine was immediately sought, leading to the initiation of empirical antimicrobial therapy with linezolid and meropenem, along with comprehensive supportive care. Despite these measures, the infection advanced aggressively, extending to the perianal region and right lower limb, threatening both bowel and motor functions. This clinical deterioration, which culminated in septic shock, heart failure, and acute renal failure, necessitated an immediate transfer to the Intensive Care Unit (ICU) and prompted an urgent multidisciplinary collaboration within 72 h of admission.

**Fig. 3:**
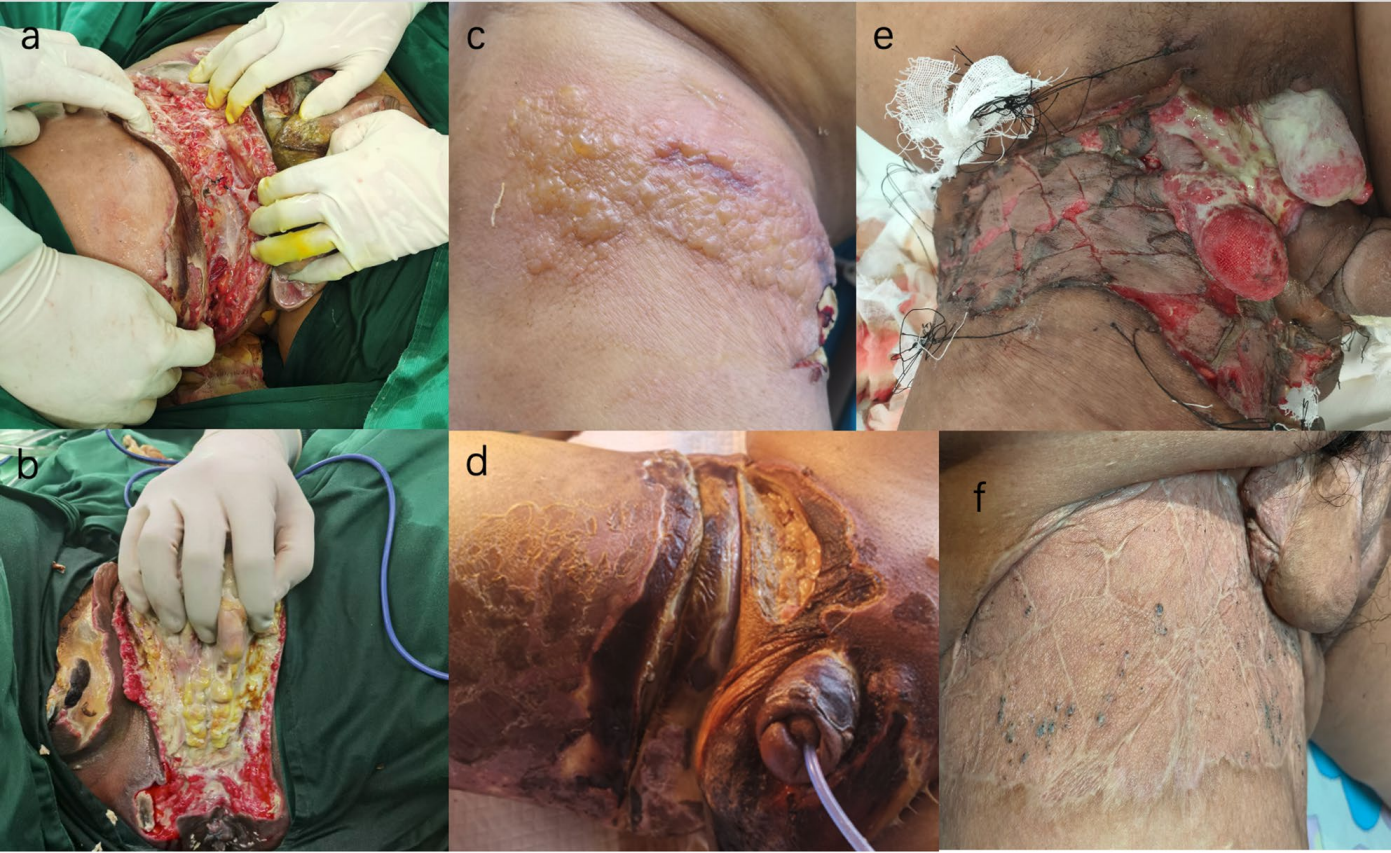
Intraoperative and postoperative pictures of the patient

**Fig. 4:**
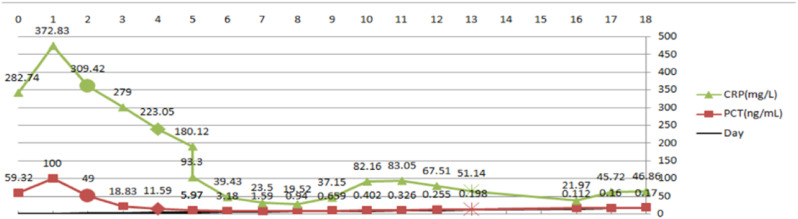
Trends of CRP and PCT levels over time. Symbols:●Empirical antibiotic phase; ◆Antibiotic change with initial debridement; ※Post-debridement phase

The Department of Laboratory Medicine confirmed *Streptococcus pyogenes* with 99.9% confidence via Matrix-Assisted Laser Desorption/Ionization Time-of-Flight Mass Spectrometry (MALDI-TOF MS), showing a susceptibility profile of penicillin sensitivity and clindamycin resistance. A follow-up CT scan demonstrated an extension of the edema to involve the pelvic wall, bilateral hips, and the scrotum. These findings, combined with the LRINEC score of 8, solidified the diagnosis of necrotizing fasciitis complicated by the aforementioned septic shock and multi-organ failure. A comprehensive therapeutic strategy was immediately implemented. The antimicrobial regimen was adjusted to high-dose intravenous penicillin G, complemented by meropenem. To address the profound systemic insult, continuous renal replacement therapy (CRRT) was initiated for renal support, and high-dose intravenous immunoglobulin (IVIG) was administered for three days. Once the patient’s hemodynamics were stabilized, the Department of Burn and Plastic Surgery performed an emergency decompressive fasciotomy. Surgical exploration confirmed the diagnosis, revealing necrotic adipose tissue with a grayish-white appearance, abundant interstitial exudate, and thrombosis within the adipose layer vessels (Fig. 3a, b). A vacuum-assisted closure (VAC) system was applied. The patient responded favorably, with inflammatory biomarkers declining markedly within two days post-operation (CRP from > 300 mg/L to 223.05 mg/L; PCT from > 100 ng/mL to 11.59 ng/mL; see serial trends in Fig. 4). However, the patient developed numerous clear, fluid-filled bullae on the right thigh, surrounding the surgical wound, resembling the appearance of severe burns (Fig. 3c). This finding provided direct evidence of dermal- fascial separation caused by the underlying infection.

The subsequent hospital course was characterized by steady recovery. A secondary debridement of necrotic scrotal skin was performed by the Department of Burn and Plastic Surgery on hospital day 11 (Fig. 3d). Following antimicrobial stewardship principles, the Pharmacy Department discontinued meropenem and de-escalated therapy to penicillin monotherapy. Simultaneously, the Nutrition Department started intensive enteral nutrition. By hospital Day 21, the patient was transferred to a tertiary hospital in a stable condition for skin grafting (Fig. 3e). At the six-month follow-up confirmed a favorable outcome, the wound had healed completely witho no residual organ dysfunction (Fig. 3f). No recurrence was reported by the patient up to manuscript submission.

## Discussion

This study reported a case of FG caused by monomicrobial GAS infection after minor trauma in a healthy adult male. Attributed to an intime systematic management by a multidisciplinary team (MDT), the patient achieved a favorable outcome. This is the first reported case in China of such extensive NF secondary minor trauma.

FG caused solely by GAS without any of these common infectious sources is rare [15]. In this case, we hopothesis that, Alcohol consumption compromised the skin barrier [16, 17], subsequent prolonged lateral positioning further impaired local microcirculation, facilitating bacterial proliferation. Furthermore, cycling-related microtrauma provided a portal for bacterial entry. Although the patient had no history of streptococcal pharyngitis and no pharyngeal cultures were obtained, we hypothesize that transient bacteremia from an asymptomatic GAS carrier state may have seeded the site of minor trauma, ultimately initiating NF.

FG typically affects immunocompromised males aged 50–70 years—such as those with HIV, immunosuppressive therapy, diabetes, or chronic alcoholism [1, 4]. In contrast, our patient is a previously healthy 37-year-old male. His initial non-specific symptoms, including localized swelling or pain, led to misdiagnosed of testicular torsion and drug-induced dermatitis. The initial treatment with parecoxib and corticosteroids may have potentially facilitate the spread of GAS infection, and their analgesic effect may mask the true severity of the condition, which may have worsened the outcome.

The LRINEC score was first introduced by Wong et al. in 2004, a score ≥ 6 carries a positive predictive value of 92% for NF [18]. In this case, laboratory results on admission yielded a LRINEC score of 8, strongly suggestive of NF; however, this finding was overlooked, contributing to a delay in definitive diagnosis. This experience reinforces the importance of routine LRINEC evaluation in all cases of suspected soft tissue infection, with high-risk scores warranting immediate surgical evaluation and advanced imaging, alongside early microbiological sampling to guide targeted antimicrobial therapy.

NF carries a mortality rate of 20–30%, and emergency surgical intervention is mandatory upon diagnosis [4]. In this case, a successful outcome was achieved through a structured MDT approach, which included timely surgical debridement as a critical component. Regarding antimicrobial therapy, although international guidelines frequently recommend combination regimens including clindamycin to suppress bacterial toxin production [4, 6], epidemiological data from China indicate high resistance rates of Group A Streptococcus (GAS) to macrolides and clindamycin, ranging from 88.9% to 100% [9, 19–21]. Therefore, our initial empirical regimen consisted of linezolid in combination with meropenem. Subsequent antimicrobial susceptibility testing confirmed that the GAS isolate was indeed resistant to clindamycin, validating our initial choice. Upon availability of susceptibility results, the regimen was adjusted to high-dose intravenous penicillin G alongside meropenem. This experience underscores that antimicrobial selection must integrate both pharmacological principles (toxin suppression) and local resistance data.

## Conclusion

This case underscores that NF can arise from seemingly minor trauma, even in immunocompetent individuals without classic risk factors. It demonstrates a unique synergistic infection pathway primarily driven by GAS. Early recognition, aided by prompt application of the LRINEC score and urgent imaging, is critical for favorable outcome. Successful management is contingent upon a time-sensitive, multidisciplinary team approach.

## Data Availability

The datasets and imaging materials supporting this case report are available from the corresponding author upon reasonable request.

## Abbreviations

NF: Necrotizing Fasciitis
FG: Fournier’s gangrene
LRINEC: Laboratory Risk Indicator for Necrotizing Fasciitis
CT: Computed Tomography
CRRT: Continuous renal replacement therapy
IVIG: Intravenous Immunoglobulin
MDT: Multidisciplinary Team
MALDI-TOF MS: Matrix-Assisted Laser Desorption/Ionization Time-of-Flight Mass Spectrometry
VAC: Vacuum-Assisted Closure
ICU: Intensive Care Unit
GAS: Group A Streptococcus
CRP: C-Reactive Protein
PCT: Procalcitonin

## References

[1] Huang RS, Patil NS, Khan Y. Periorbital necrotizing fasciitis: case presentation. Interact J Med Res. 2023;12:e52507. 37971729 10.2196/52507 PMC10716763

[2] Zhu SB, Li DH, Shuntang L, et al. Analysis of clinical characteristics and treatment of patients with perianal necrotizing fasciitis. Chin J Burns Wounds. 2024:955–62.

[3] Chernyadyev SA, Ufimtseva MA, Vishnevskaya IF, et al. Fournier’s gangrene: literature review and clinical cases. Urol Int. 2018;101:91–7. 29949811 10.1159/000490108 PMC6106138

[4] Wöhler A, Schwab R, Güsgen C, et al. [Diagnosis and treatment of severe fournier’s gangrene: introduction of a surgical Approach, evaluation of risk Factors, Microbiological characteristics and review of the Literature]. Zentralbl Chir. 2022;147:480–91. 33556981 10.1055/a-1319-1734

[5] Brébant V, Eschenbacher E, Hitzenbichler F, Pemmerl S, Prantl L, Pawlik M. Pathogens and their resistance behavior in necrotizing fasciitis. Clin Hemorheol Microcirc. 2024;86:169–81. 37807775 10.3233/CH-238119

[6] Pérez-Sánchez I, Martínez-Gil L, Piqueras-Vidal PM, Pont-Gutiérez C, Cebrián-Gómez R, Montoza-Nuñez JM. [Translated article] necrotising fasciitis: management experience over the last two decades in our hospital. Rev Esp Cir Ortop Traumatol. 2022;66:T11–9. 35853609 10.1016/j.recot.2021.12.009

[7] Tam PCK, Kennedy B, Ashokan A. Necrotizing soft tissue infections in South australia: A 15-Year review. Open Forum Infect Dis. 2023;10:ofad117. 37035499 10.1093/ofid/ofad117 PMC10077820

[8] Zhang KF, Shi CX, Chen SY, Wei W. Progress in multidisciplinary treatment of fournier’s gangrene. Infect Drug Resist. 2022;15:6869–80. 36465810 10.2147/IDR.S390008 PMC9717591

[9] Wang J, Ma C, Li M, et al. Streptococcus pyogenes: pathogenesis and the current status of vaccines. Vaccines (Basel). 2023;11. 10.3390/vaccines11091510 PMC10534548 37766186

[10] Brouwer S, Rivera-Hernandez T, Curren BF, et al. Pathogenesis, epidemiology and control of group A Streptococcus infection. Nat Rev Microbiol. 2023;21:431–47. 36894668 10.1038/s41579-023-00865-7 PMC9998027

[11] Salati SA. Necrotizing fasciitis a review. Pol Przegl Chir. 2022;95:1–8. 36805313 10.5604/01.3001.0015.7676

[12] Stevens DL, Bisno AL, Chambers HF, et al. Practice guidelines for the diagnosis and management of skin and soft tissue infections: 2014 update by the infectious diseases society of America. Clin Infect Dis. 2014;59:e10–52. 24973422 10.1093/cid/ciu444

[13] Wu PH, Wu KH, Hsiao CT, Wu SR, Chang CP. Utility of modified laboratory risk indicator for necrotizing fasciitis (MLRINEC) score in distinguishing necrotizing from non-necrotizing soft tissue infections. World J Emerg Surg. 2021;16:26. 34039397 10.1186/s13017-021-00373-0 PMC8157441

[14] [Chinese expert consensus on diagnosis and treatment of perianal necrotizing fasciitis. (2019)]. Zhonghua Wei Chang Wai Ke Za Zhi. 2019;22:689–93. 10.3760/cma.j.issn.1671-0274.2019.07.017 31302971

[15] El-Qushayri AE, Khalaf KM, Dahy A, et al. Fournier’s gangrene mortality: A 17-year systematic review and meta-analysis. Int J Infect Dis. 2020;92:218–25. 31962181 10.1016/j.ijid.2019.12.030

[16] van der Heide FCT, Eussen S, Houben A, et al. Alcohol consumption and microvascular dysfunction: a J-shaped association: the Maastricht study. Cardiovasc Diabetol. 2023;22:67. 36964536 10.1186/s12933-023-01783-x PMC10039613

[17] Liu L, Chen J. Advances in relationship between alcohol consumption and skin diseases. Clin Cosmet Investig Dermatol. 2023;16:3785–91. 38169933 10.2147/CCID.S443128 PMC10759914

[18] Powell LM, Choi SJ, Chipman CE, Grund ME, LaSala PR, Lukomski S. Emergence of Erythromycin-Resistant invasive group A Streptococcus, West Virginia, USA, 2020–2021. Emerg Infect Dis. 2023;29:898–908. 37080963 10.3201/eid2905.221421 PMC10124663

[19] Zhao HL, Zhao XH, Yang B, Shi M, Sun ZG. [Comprehensive treatment of 25 cases of acute necrotizing fasciitis]. Zhonghua Shao Shang Za Zhi. 2021;37:382–5. 33887885 10.3760/cma.j.cn501120-20200426-00238 PMC11917329

[20] Zhang Yinghua HY, Wang Xiaoguang HY, Hongjing Y. Molecular epidemiological characteristics of streptococcus pyogenes causing Scarlet fever and angina in children. Chin J Microbiol Immunol. 2019;(11):821–6.

[21] Li X-HFYCX. Zhang Yan-ling, Zhang Na. Distribution and drug resistance analysis of pathogenic bacteria in blood stream infection of hospitalized patients in a hospital from 2017 to 2022. World Notes Antibiot. 2024;45(05):312–8.

